# Racial and Ethnic Disparities in Primary Open-Angle Glaucoma Clinical Trials: A Systematic Review and Meta-analysis

**DOI:** 10.1001/jamanetworkopen.2021.8348

**Published:** 2021-05-18

**Authors:** Karen Allison, Deepkumar G. Patel, Leah Greene

**Affiliations:** 1 New York Ophthalmology Associates, Manhattan; 2 School of Health Sciences and Practice & Institute of Public Health, New York Medical College, Valhalla

## Abstract

**Importance:**

The disease burden for primary open-angle glaucoma (POAG) is highest among racial/ethnic minority groups, particularly Black individuals. The prevalence of POAG worldwide is projected to increase from 52.7 million in 2020 to 79.8 million in 2040, a 51.4% increase attributed mainly to Asian and African individuals. Given this increase, key stakeholders need to pay particular attention to creating a diverse study population in POAG clinical trials.

**Objective:**

To assess the prevalence of racial/ethnic minorities in POAG clinical research trials compared with White individuals.

**Data Sources:**

This meta-analysis consisted of publicly available POAG clinical trials using ClinicalTrials.gov, PubMed, and Drugs@FDA from 1994 to 2019.

**Study Selection:**

Randomized clinical trials that reported on interventions for POAG and included demographic subgroups including sex and race/ethnicity.

**Data Extraction and Synthesis:**

Using the Preferred Reporting Items for Systematic Reviews and Meta-Analyses (PRISMA) guidelines, 2 independent reviewers extracted study-level data for a random-effects meta-analysis. A third person served as the tiebreaker on study selection. Microsoft Excel 2016 (Microsoft Corporation) and SAS, version 9.4 (SAS Institute) were used for data collection and analyses.

**Main Outcomes and Measures:**

The primary outcomes were the prevalence of each demographic subgroup (White, Black, Hispanic/Latino, other race/ethnicity groups, and female or male) in each trial according to the trial start year, study region, and study sponsor. Participation rates are expressed as percentages.

**Results:**

A total of 105 clinical trials were included in the meta-analysis, including 33 428 POAG clinical trial participants (18 404 women [55.1%]). Overall, 70.7% were White patients, 16.8% were Black patients, 3.4% were Hispanic/Latino patients, and 9.1% were individuals of other races/ethnicities, including Asian, Native Hawaiian or Pacific Islander, American Indian or Alaska Native, and unreported as defined by the US Census. The mean (SD) numbers of participants by race/ethnicity were 236.5 (208.2) for White, 58.4 (70.0) for Black, 29.9 (71.1) for Hispanic/Latino, and 31.1 (94.3) for other race/ethnicity. According to the test for heterogeneity using the Cochrane Risk of Bias tool, the *I*^2^ statistic was 98%, indicating high heterogeneity of outcomes in the included trials. A multiple linear regression analysis was performed to assess any trend and significance between participation by Black individuals and the year the study started, the region in which the study took place, and the study sponsor. There was no significant increase of Black participant enrollment from 1994 to 2019 (*r*^2^ = 0.11; *P* = .17) and no significant association between Black participant enrollment and clinical trial region (*r*^2^ = 0.16; *P* = .50), but there was a significant association between Black participant enrollment and study sponsor (*r*^2^ = 0.94; *P* = .03).

**Conclusions and Relevance:**

This meta-analysis found that compared with White individuals, individuals from racial/ethnic minority groups had a very low participation rate in POAG clinical trials despite having a higher prevalence among the disease population. Despite measures to increase clinical trial diversity, there has not been a significant increase in clinical trial participation among Black individuals, the group most affected by this disease; this disparity in POAG clinical trial representation can raise questions about the true safety and efficacy of approved medical interventions for this disease and should prompt further research on how to increase POAG clinical trial diversity.

## Key Points

**Question:**

What is the prevalence of participants from racial/ethnic minority groups compared with White individuals in primary open-angle glaucoma (POAG) clinical trials?

**Findings:**

In this meta-analysis of 33 428 POAG clinical trial participants, 70.7% of the study population was White, 16.8% was Black, 3.4% was Hispanic/Latino, and 9.1% consisted of other races/ethnicities, including Asian, Native Hawaiian or Pacific Islander, American Indian or Alaska Native, and unreported. The proportion of Black participants did not significantly increase from 1994 to 2019, but there was a significant association between study sponsor and participation of Black individuals in clinical trials.

**Meaning:**

Results suggest that racial/ethnic minority groups have a significantly lower participation rate in POAG clinical trials compared with White individuals despite having a higher disease burden.

## Teaser

This meta-analysis investigates the prevalence of participants from racial/ethnic minority groups compared with White individuals in clinical trials for primary open-angle glaucoma.

## Introduction

Glaucoma is a group of eye conditions that damage the optic nerve, causing blindness and visual impairment, affecting approximately 52.7 million people in 2020, and the prevalence is projected to increase to 79.8 million in 2040. The 51.4% increase is attributed mainly to individuals in Asia and Africa.^1^ As the leading cause of irreversible blindness worldwide, glaucoma has been reported to be 7 times more likely to cause blindness in Black individuals compared with White individuals and 15 times more likely to cause visual impairment among Black individuals compared with White individuals.^2,3,4^ Primary open-angle glaucoma (POAG) is the most common form of the disease and currently affects approximately 8.73 million people of African ancestry worldwide.^1,4,5,6,7,8^ In the US in 2010, Black individuals had the highest prevalence rate of POAG at 3.4% compared with White individuals at 1.7%.^9^ Primary open-angle glaucoma occurs when the angle between the cornea and iris is open and the drainage system slowly becomes clogged over time, causing increased pressure on the optic nerve.^4,10,11,12,13^ This initially leads to a decrease in peripheral vision and eventually central vision, resulting in complete vision loss.^4,13^

Age and race/ethnicity have been reported to be associated with POAG. Previous observational studies of genetic variants associated with POAG among individuals of African descent compared with those of European descent have been inconclusive in elucidating the genetics of POAG in African American individuals and other individuals of African descent .^12^ Further research is needed to gain a better understanding of the mechanism of the disease and the possible role of genetics in increasing the risk of developing POAG. Currently, POAG can be treated if detected early, but there is no treatment that can restore vision once it has been lost. Medical interventions are increasingly likely to be safer and more effective for all with a diverse pool of clinical trial participants; thus, there has been a growing concern about the lack of diversity among clinical research populations by race and ethnicity.

Since the 1990s, the US Food and Drug Administration (FDA) has implemented several strategies to encourage greater participation among minority communities, most notably the FDA Safety and Innovation Act of 2012. Section 907 of this law requires the FDA to examine the quality of demographic subgroup data, the diversity of participants by subgroup, and the transparency and availability of access to these demographic subgroup data.^14,15^ The use of eye care services for age-related diseases decreases as socioeconomic disadvantages increase.^16^ Socioeconomic status is a social determinant of health status, and racial/ethnic minority groups experience more socioeconomic disadvantages compared with White individuals.^17^ In 2008, 52.6% of non-Hispanic White individuals self-reported annual eye care visits, whereas only 47.2% of non-Hispanic Black individuals and 36.9% of Hispanic individuals self-reported annual eye care visits.^17^ Individuals with a poverty income ratio of 1.00 were less likely to have an annual eye care visit than those with a poverty income ratio of 4.00.^17^ Access to adequate health care is related to lack of diversity among participants in glaucoma clinical trials. To our knowledge, very few studies exist that address any changes in participant diversity among POAG clinical trials. Consequently, we investigated racial/ethnic disparities among these trials since 1994. In this study, our goal was to conduct a meta-analysis of demographic data from completed, interventional POAG clinical trials with results using publicly available data to analyze the prevalence of racial/ethnic minority participants among these study populations.

## Methods

This meta-analysis included all studies from 1994 to 2019 and was deemed exempt from institutional review board approval and informed consent because it collected and synthesized nonidentifiable data from previously published studies. A systematic review was conducted using a meta-analysis of clinical studies following the Preferred Reporting Items for Systematic Reviews and Meta-analyses (PRISMA) reporting guideline.^18^ Literature and clinical studies were searched and reviewed using ClinicalTrials.gov, PubMed, and the FDA website using Drugs@FDA including only English-language publications from 1994 to 2019. Keywords used for this search on ClinicalTrials.gov included *glaucoma, primary open angle*, *studies with results*, and *interventional studies (clinical trials)*. Keywords used for this search on PubMed included *primary open-angle glaucoma* and *therapy*.

### Inclusion and Exclusion Criteria and Data Extraction

Studies were included if they met the following criteria: (1) POAG clinical trials, (2) completed trials, (3) interventional studies, (4) publicly available studies with results, (5) institutional review board–approved studies, and (6) demographic subgroups including sex/gender and race/ethnicity. Studies were excluded that did not include (1) race/ethnicity, (2) sex or gender, or (3) an intervention; or that were (4) still ongoing, (5) still open to accrual, or (6) not published in English.

We extracted data including (1) medical intervention, (2) number of participants, (3) year the study started, (4) year the study ended, (5) region in which the studies were conducted, (6) study sponsor, (7) participant sex/gender, (8) ethnicity (if indicated), and (9) race. The geographic region of a study was defined using the United Nations classification, which includes Africa, Asia, Europe, Latin America and the Caribbean, North America, and Oceania.^19^ We also added “multiregional” as a category to account for studies that encompassed more than 1 region. Race was captured using 3 categories: (1) White, (2) Black, and (3) other; ethnicity was recorded as Hispanic/Latino, if reported. Racial subgroups included as “other” consisted of Asian, Native Hawaiian or Pacific Islander, American Indian or Alaska Native, and unreported as defined by the US Census.^20^ Sponsors for these trials were US-based, non–US-based, or not a pharmaceutical company and were organized as such into (1) US pharmaceutical company, (2) non-US pharmaceutical company, (3) nonpharmaceutical company, and (4) collaborator, which included pharmaceutical and nonpharmaceutical companies together, because some sponsors of more than 1 category worked together on 1 trial. Details on the sponsors for each of the trials are listed in Table 1.

**Table 1.** Participant Demographic Characteristics in POAG Clinical Trials Since 1994

Variable	No. of trials	Total participants, No.	Participants, No. (%)					
			Race/ethnicity				Sex	
			White	Black	Other	Hispanic/Latino	Female	Male
Trial start year								
1994	1	1636	1138 (69.6)	407 (24.9)	32 (2.0)	59 (3.6)	931 (56.9)	705 (43.1)
1996	3	828	736 (88.9)	74 (8.9)	2 (0.2)	16 (1.9)	408 (49.3)	420 (50.7)
1997	1	96	76 (79.2)	11 (11.5)	0	9 (9.4)	52 (54.2)	44 (45.8)
1998	6	1728	1274 (73.7)	210 (12.2)	117 (6.8)	127 (7.3)	906 (52.4)	822 (47.6)
2001	14	5088	3963 (77.9)	677 (13.3)	175 (3.4)	273 (5.4)	2680 (52.7)	2408 (47.3)
2003	1	282	224 (79.4)	4 (1.4)	54 (19.1)	0	138 (48.9)	144 (51.1)
2005	5	1318	871 (66.1)	122 (9.3)	294 (22.3)	31 (2.4)	709 (53.8)	609 (46.2)
2006	1	516	465 (90.1)	27 (5.2)	24 (4.7)	0	243 (47.1)	273 (52.9)
2007	1	178	168 (94.4)	3 (1.7)	5 (2.8)	2 (1.1)	90 (50.6)	88 (49.4)
2009	6	826	673 (81.5)	106 (12.8)	25 (3.0)	22 (2.7)	430 (52.1)	396 (47.9)
2010	6	2423	1819 (75.1)	387 (16.0)	59 (2.4)	158 (6.5)	1321 (54.5)	1102 (45.5)
2011	9	2686	1624 (60.5)	537 (20)	466 (17.3)	59 (2.2)	1607 (59.8)	1079 (40.2)
2012	8	930	650 (69.9)	181 (19.5)	72 (7.7)	27 (2.9)	473 (50.9)	457 (49.1)
2013	9	1647	1056 (64.1)	258 (15.7)	235 (15.7)	98 (6.0)	932 (56.6)	715 (43.4)
2014	12	4256	3085 (72.5)	938 (22.0)	133 (3.1)	100 (2.3)	2383 (56)	1873 (44)
2015	8	4109	2457 (59.8)	760 (18.5)	872 (21.2)	20 (0.5)	2255 (54.9)	1854 (45.1)
2016	6	1369	960 (70.1)	361 (26.4)	7 (0.5)	41 (3.0)	799 (58.4)	570 (41.6)
2017	6	2044	1422 (69.6)	354 (17.3)	99 (4.8)	169 (18)	1230 (60.2)	814 (39.8)
2019	2	1468	975 (66.4)	195 (13.3)	53 (3.6)	245 (16.7)	855 (58.2)	613 (41.8)
Region								
Asia	3	537	0	0	537 (100.0)	0	293 (54.6)	244 (45.4)
Europe	15	3519	3222 (91.6)	194 (5.5)	1 (0.03)	102 (2.9)	1439 (40.9)	2080 (59.1)
Latin America	2	187	111 (63.4)	51 (29.1)	2 (1.1)	23 (12.2)	116 (62.0)	71 (38.0)
North America	76	24 813	17 738 (71.5)	4975 (20.0)	2000 (8.1)	100 (0.4)	14 508 (58.5)	10 305 (41.5)
Multiregional	9	3242	2565 (79.1)	392 (12.1)	95 (2.9)	190 (5.9)	1877 (57.9)	1365 (42.1)
Sponsor								
US pharmaceutical	40	16 153	11 481 (71.1)	2738 (17)	1735 (10.7)	199 (1.2)	9161 (56.7)	6992 (43.3)
Non-US pharmaceutical	48	13 924	10 096 (72.5)	2218 (15.9)	1410 (10.1)	200 (1.4)	7629 (54.8)	6295 (45.2)
Nonpharmaceutical	14	2690	1881 (70.0)	567 (21.1)	215 (8.0)	27 (1.0)	1364 (50.7)	1326 (49.3)
Collaborators	3	555	272 (49.0)	90 (16.2)	193 (34.8)	0	132 (23.8)	423 (76.2)

Abbreviation: POAG, primary open-angle glaucoma.

### Statistical Analysis

Statistical analyses were conducted using Microsoft Excel 2016 (Microsoft Corporation) and SAS software, version 9.4 (SAS Institute, Inc). Demographic data from the study population were collected, and participation by sex/gender, race, and ethnicity of each trial was calculated as a percentage of total participants in the study. Trials were grouped according to the trial start year, the region in which the trial took place, and the type of study sponsor. A multiple linear regression was performed to investigate a trend in Black participation by each independent variable. The independent variables for this analysis were the year each study started, the region, and study sponsor, and the dependent variable was the number of Black clinical trial participants. All assumptions were met to conduct a multiple linear regression analysis. There was a linear association between participation by Black individuals and all independent variables, the residuals were normally distributed, and the independent variables were not highly correlated with each other. Descriptive statistics were obtained for all collected data. A 1-way analysis of variance was performed to assess the significance of the means for more than 2 groups, and then an independent-samples *t* test was used to assess the differences between 2 groups and correct for multiplicity. Hypothesis tests were 2-sided. Internal validity of the included studies was analyzed using the Cochrane Risk of Bias tool. The *I*^2^ statistic was 98%, indicating high heterogeneity among the study outcomes. *P* ≤ .05 was considered statistically significant for all analyses.

## Results

### Study Selection Process

The study selection process that was used in this meta-analysis is depicted in the eFigure in the Supplement.^18^ A total of 1797 studies were initially identified through a comprehensive search of randomized clinical trials using PubMed, ClinicalTrials.gov, and Drugs@FDA. Subsequently, 868 studies were removed as duplicates, leaving 929 potentially eligible clinical trials. A total of 680 studies were then excluded based on keywords from ClinicalTrials.gov and PubMed, leaving 249 potentially eligible clinical trials. A final set of 144 studies was excluded based on inclusion and exclusion criteria. Studies were excluded owing to the lack of available demographic subgroup data that included race.

Ultimately, 105 clinical trials were included in the meta-analysis, including 33 428 POAG clinical trial participants (18 404 women [55.1%]; 23 636 White participants [70.7%]) (eFigure in the Supplement). Three authors (K.A., D.G.P., L.G.) were involved in the study selection process. There were 2 reviewers (D.G.P., L.G.), and the third author (K.A.) served as the tiebreaker on study selection. Search terms used in the study selection process can be found in eTable 1 in the Supplement.

### General Characteristics of Included Studies and Participants

Characteristics of glaucoma treatments in these trials are listed in eTable 2 in the Supplement. Drug trials encompassed 89 of 105 (85%) of the reviewed trials and 91% of the total study population. Additionally, there were more trials that evaluated the safety and efficacy of devices; however, there were more members of the total study population among procedure-based trials.

The total numbers of participants along with demographic data extracted from each study by the trial start year, region, and type of study sponsor are displayed in Table 1. The region of each study is based on the United Nations classification of geographic region^19^; however, Africa, the Caribbean, and Oceania were excluded from this table because clinical trials did not take place exclusively in those regions. They were, however, included among 6 multiregional studies (Table 1).

**Figure 1.**
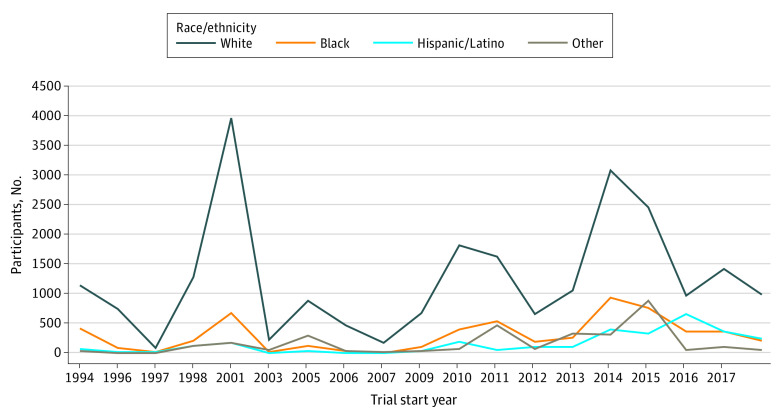
Race/Ethnicity Trends by Trial Start Year

Figure 1 compares the overall numbers of participants enrolled in the trials by race and ethnicity according to the trial start year based on the data from Table 1. Trial participation was evaluated in terms of trial start year in order to depict any trends in trial participation by race and ethnicity at the time of enrollment. Some years had very few trials compared with others, but including all years that trials were conducted from 1994 to 2019 was important for our analysis in order to evaluate for increases in participation by Black individuals during this time period.

Figure 1 shows that White participants composed 70.7% of the total study population. Black participants made up only 16.8% of the total trial participation. Hispanic/Latino individuals comprised 3.4% of participants, and individuals categorized as other comprised 9.1% of participants. There was a steady increase in participation by minority racial/ethnic groups after 2003; Black participants, Hispanic/Latino participants, and those categorized as other did not surpass 1000 participants individually as their own subgroup at any year (Figure 1). Only 2 trials contained more Black participants than White participants. In these 2 trials, which were conducted in North America, 56.3% were Black participants and 40.0% were White participants.

The largest number of White participants occurred in 2001, with 3963 of 5088 (77.9%), whereas the number of Black participants at that time was 677 of 5088 (13.3%). The year 2014 contained clinical trials with the largest number of Black participants at 938 of 4256 (22%), with White participants at 3085 of 4256 (72.5%).

**Figure 2.**
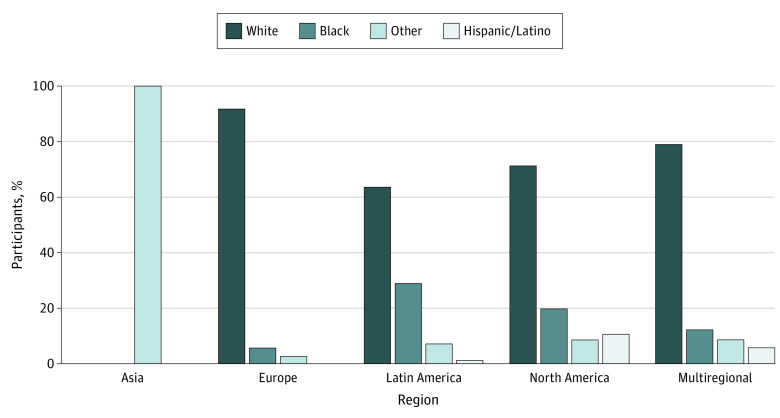
Race/Ethnicity Trends by Region

Figure 2 compares the overall percentage of participants by race and ethnicity enrolled in the trials according to geographic region, based on data from Table 1. White participants were the largest racial subgroup in Europe, Latin America, North America, and multiregion studies. In each region except Asia, White individuals comprised no less than 63.4% of the total trial participation, whereas Black individuals comprised no more than 29.1% of the trial participation. Hispanic/Latino participants comprised no more than 12.2% of participants. There were 3 trials that took place in Asia that involved participants who had exclusively reported Asian as their race/ethnicity. The participants in those trials were categorized as “other.”

**Figure 3.**
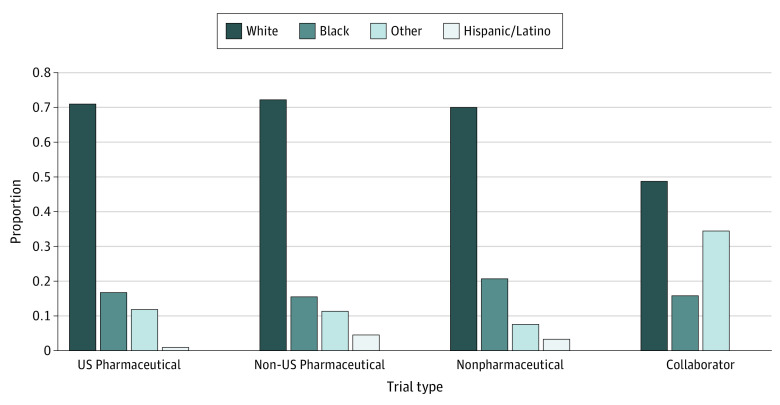
Race/Ethnicity by Study Sponsor

Figure 3 compares the overall proportions of participants by race and ethnicity according to the type of study sponsor based on data from Table 1. Non-US pharmaceutical companies had the highest White participation rate (10 096 of 13 924 [72.5%]), whereas US pharmaceutical companies had the highest raw numbers of White participants (11 481). On the other hand, US pharmaceutical companies had the highest raw numbers of Black participants (2738), whereas nonpharmaceutical companies had the highest participation rate for Black individuals (567 of 2690 [21.1%]). Individuals in the other race/ethnicity group had a higher prevalence of participation (193 of 555 [34.8%]) in trials with nonpharmaceutical and pharmaceutical sponsors that worked collaboratively compared with Black individuals (90 of 555 [16.2%]).

### Trend in Participation of Black Individuals in POAG Clinical Trials

The prevalence of Black participants among the total study population by the trial start year, region, and study sponsor is displayed in Table 2. The total population of Black people in these trials was 5612 of 33 428, accounting for only 16.8% of the total study population. In 2014, participation of Black individuals in these trials reached its peak at 938 of 4256 participants (22.0%), but it declined steadily in the years that followed. During those years, however, the number of clinical trials decreased as well, which may contribute to the decrease in participation by Black individuals. In 2016, the participation rate for Black patients was at its highest at 361 of 1369 (26.4%) of the total trial population. In 2007, there was only 1 clinical trial with only 3 Black participants, who made up 1.7% of the 178 participants, and 168 White participants, who made up 94.4% of the 178 participants enrolled. The enrollment of Black participants did not significantly increase from 1994 to 2019 (*r*^2^ = 0.11; *P* = .17) (Table 2).

**Table 2.** Black Participants in POAG Clinical Trials

Variable	No. of trials	Total population	Black participants, No. (%)	*P* value
Trial start year				
1994	1	1636	407 (24.9)	.17^a^
1996	3	828	74 (8.9)	
1997	1	96	11 (11.5)	
1998	6	1728	210 (12.2)	
2001	14	5088	677 (13.3)	
2003	1	282	4 (1.4)	
2005	5	1318	122 (9.3)	
2006	1	516	27 (5.2)	
2007	1	178	3 (1.7)	
2009	6	826	106 (12.8)	
2010	6	2423	387 (16.0)	
2011	9	2686	537 (20)	
2012	8	930	181 (19.5)	
2013	9	1647	258 (15.7)	
2014	12	4256	938 (22.0)	
2015	8	4109	760 (18.5)	
2016	6	1369	361 (26.4)	
2017	6	2044	354 (17.3)	
2019	2	1468	195 (13.3)	
Region				
Asia	3	537	0	.50^a^
Europe	15	3519	194 (5.5)	
Latin America	2	187	51 (29.1)	
North America	76	24 813	4975 (20.0)	
Multiregional	9	3242	392 (12.1)	
Sponsor				
US pharmaceutical	40	16 153	2738 (17)	.03^a^
Non-US pharmaceutical	48	13 924	2218 (15.9)	
Nonpharmaceutical	14	2690	567 (21.1)	
Collaborators	3	555	90 (16.2)	

Abbreviation: POAG, primary open-angle glaucoma.

^a^ Multiple linear regression used.

The number of Black participants was highest in North America at 4975 of 24 813 participants (20%) and lowest in Asia with no participation, but North America contained the most clinical trials. The rate of participation by Black individuals was highest in Latin America at 29.1%, although the overall number of participants was lower, with 51 Black participants of 187 in total. There was no statistically significant difference (*r*^2^ = 0.16; *P* = .50) in representation of Black participants by region of the clinical trials.

In terms of the type of study sponsor, participation by Black individuals was highest among studies funded by US pharmaceutical companies at 2738 participants of 16 153, but nonpharmaceutical companies had the highest participation rate of Black individuals at 21.1%. There was a statistically significant association between Black participant representation and the type of study sponsor (*r*^2^ = 0.94; *P* = .03). All study sponsors included in this meta-analysis can be found in eTable 3 in the Supplement.

## Discussion

The results of our meta-analysis of 105 POAG clinical trials with 33 428 participants suggest that individuals from racial/ethnic minority groups, especially Black individuals, had a lower participation rate than that of White individuals despite making up a larger proportion of people with the disease. From 1994 to 2019, there was no significant evidence to suggest that there had been a considerable increase in study participation by minority groups despite tangible steps taken to minimize this disparity. In total, White individuals made up 70.7% of the study population, whereas Black individuals made up only 16.8%, Hispanic/Latino individuals made up 3.4%, and those categorized as other made up 9.1%.

### Lack of Black Participant Representation

The majority of the clinical trials included primarily White participants except for 2 trials^21,22^ that consisted of more Black participants. It is estimated that the global prevalence of POAG is 3.05%, with an odds ratio of 2.88 (95% CI, 1.97-4.10) for individuals of African ancestry compared with individuals of European ancestry.^1^ White participants from our analysis outnumbered Black participants by approximately 4:1, whereas the disease burden for POAG in 2010 was 1:2 for White to Black participants.^9^ This finding coincides with other study findings that representation of racial/ethnic minority groups is lacking in clinical trials^6,23^ and that stricter requirements are needed to ensure that diverse pools of participants are being included in POAG clinical trials.

### Importance of a Representative Population

To our knowledge, there has been very little research conducted to explore the effects of glaucoma medical interventions stratified by race and ethnicity. From what has been done, investigators concluded that more research needs to be conducted to make any definitive assumptions on the implications of race and ethnicity for glaucoma medication response. The Prostaglandin Efficacy and Safety Study Undertaken by Race (PRESSURE) study,^24^ which examined the safety and efficacy of prostaglandin, a common type of treatment for POAG, found that there were no ethnicity–based differences in the ability of the drug to lower intraocular pressure, which assists in minimizing the effects of POAG. However, the authors of the study indicated that their sample size did not have enough power to detect a difference and stressed the need to conduct similar studies with larger sample sizes.^24^

Although the research on race/ethnicity-based differences in the safety and efficacy of POAG treatments is minimal, there is a plethora of research that concludes that there is a clinical and ethical need to conduct research with a representative population that is generalizable to the target population. To achieve this broader representation, future investigators need to diversify their study populations to include more participants from minority groups, especially with the implementation of pharmacogenomics and personalized medicine. A generalizable population supports implications for future research and conclusions to be drawn about the target population. Two ways to create generalizable results are making sure observations are being drawn from the proper disease population and achieving diversity in the study population so as to increase the likelihood of being comparable with the population of interest.^25,26^ Although race is a social construct,^27^ researchers also agree that race and ethnicity need to be included in the analysis of the pharmacogenetics of any drug as a socioethical responsibility and an important step in the development of personalized medicine.^28^

### Overcoming the Challenge to Increase Clinical Trial Diversity

Diversity in clinical trials is imperative to create safe and effective treatments for everyone, yet there are several challenges to increasing participant diversity. Common barriers for minority populations include mistrust, an inability to commit because of time and resource constraints, limited education and awareness of clinical trials, and lack of diversity in primary investigators.^23^ Glaucoma requires regular follow-up of patients that leads to confidence and receptiveness to physician guidance. With glaucoma, there is a lack of information and education regarding the irreversibility of vision loss and the need for lifelong treatment.^29^ There is also an economic burden: In 2013 in the US, individuals with vision impairment incurred $15 900 in annual medical expenses, and those who were blind incurred $26 900.^30^ There is a misconception, however, that people in racial and ethnic minority groups do not wish to participate in clinical trial research owing to prior implications of unethical studies, such as the Tuskegee syphilis study.^31^ Initial resistance to enrollment in a clinical trial developing from mistrust is one of many reasons why people from minority groups, specifically Black individuals, are hesitant to participate, but there is no significant evidence to suggest that minority groups refuse to participate in health research compared with non-Hispanic White individuals from mistrust alone.^31^

Steps have been taken over the years to increase participant diversity. The FDA Safety and Innovation Act indicates the necessity of including demographic subgroups by sex, age, race, and ethnicity in clinical trials to ensure that investigators and sponsors are held accountable for diversifying their study population.^14,15,32^ Transparency and access to data are required so that the FDA can assess progress in diversity and inclusion of clinical trial participants.^14,15^ The implementation of this law may be an explanation for the increase in minority participation in 2014 (Figure 1). In 2019, the FDA released detailed guidelines on how to effectively expand participant diversity, such as broadening eligibility criteria, expanding access, creating a trial that is less onerous for participants, and adopting enrollment practices that will retain inclusivity and diversity.^32^ The National Institutes of Health has also contributed to increasing diversity among clinical trials by releasing guidelines in 2017 requiring that researchers conducting National Institutes of Health–defined phase 3 clinical trials include sex, race, and/or ethnicity so that an analysis can be done to assess whether the variable or drug of interest affects women and minority participants differently.^33^

Key stakeholders involved in clinical trials are extremely important when recruiting for clinical trials. Referring physicians, study investigators, and sponsors each have a direct hand in who is allowed to enroll in a clinical trial and in overall study design.^23^ Lack of trust and limited education and clinical trial awareness are some relevant challenges to minority clinical trial participation; therefore, a referring physician serves as a trustworthy source for patients who would benefit from clinical trial participation and can educate them on the clinical benefits.^23^ Study investigators can assist by recognizing and facilitating support measures for patients who may experience time and resource constraints. Additionally, investigators can assist with transparency regarding the participants’ disease, educate them on the clinical benefits of the trial and how it will improve their quality of life, and emphasize the importance of regular follow-up and adhering to protocol standards.^23,29^ Finally, the sponsor has an obligation to stress the importance of the diversity of the study population, as this would assist their analysis of the safety and efficacy of a study drug for a representative population, thereby abiding by FDA guidelines for study drug approval.^23^

### Limitations

There are limitations to our study. First, we did not extract age from the trials we included in our meta-analysis, which is relevant to the severity of POAG for all affected individuals regardless of race/ethnicity. All studies chosen for this review included patients older than 40 years; however, the severity of POAG and age have a directly proportional relationship.^3,5,7,11,12^ Not all of the included studies reported age in their results. Second, a majority of the studies reviewed were from the North American region alone, particularly from the US, which may not be entirely representative of the disease population globally. This limitation is likely related to the fact that data were extracted only from English-language publications. Africa, the Caribbean, and Oceania did not have any trials exclusively done in those regions, and it is likely that only choosing English publications could have had an effect on our lack of clinical trial data from those regions exclusively. Third, we excluded trials that were still ongoing and open to accrual. In future meta-analyses, it should be a point of interest to study the prevalence of minorities in these types of trials. Last, our chosen studies did not always report ethnicity. Only 75 studies reported whether patients were Hispanic/Latino, which means that the prevalence of this demographic group could be underrepresented in the meta-analysis. This limitation is representative of the disparity that is present in POAG clinical trials, as the Hispanic/Latino population is also greatly affected by this disease. Some of the trials included in this study included demographic descriptors that only allowed a participant to identify with 1 category and offered no insight into whether these participants considered themselves to be a part of more than 1 demographic subgroup. For future research, it is important to include this information in the study design and enrollment process in the analysis of these trials, as it may be pertinent to a participant’s response to an intervention and future public health implications.

## Conclusions

The results of this meta-analysis suggest that there was a substantial difference between participation of members of racial/ethnic minority groups and White individuals in the POAG trials analyzed between 1994 and 2019. Despite measures taken to increase clinical trial diversity, racial/ethnic disparities among POAG clinical trial participants still persist. Individuals in minority groups, particularly those of African ancestry, are disproportionately affected by POAG yet are still inadequately represented in interventional clinical trials. This deficit in minority participation does not accurately represent the disease population. Therefore, it is crucial that new POAG clinical trials strive to meet the expectations of involving a representative population for any medical intervention that is pending approval. Efforts by the FDA, the National Institutes of Health, and stakeholders are necessary to enhance clinical trial diversity. We encourage future studies to identify any barriers that may limit minority representation and to proactively work to establish strategies to promote inclusivity and a representative population.

## Notes

Supplement. **eFigure.** Study Selection Process Using the PRISMA Format **eTable 1.** Search Terms Used **eTable 2.** Characteristics of Glaucoma Treatments in POAG Clinical Trials **eTable 3.** Types of Sponsors
