# Supplementary material for: Racial and Ethnic Disparities in Primary Open-Angle Glaucoma Clinical Trials: A Systematic Review and Meta-analysis
Source: JAMA Netw Open. 2021 May 18;4(5):e218348. doi: 10.1001/jamanetworkopen.2021.8348 (PMC8132140; doi:10.1001/jamanetworkopen.2021.8348)
Supplement: Supplement. — eFigure. Study Selection Process Using the PRISMA Format eTable 1. Search Terms Used eTable 2. Characteristics of Glaucoma Treatments in POAG Clinical Trials eTable 3. Types of Sponsors [file jamanetwopen-e218348-s001.pdf]

# Supplementary Online Content

Allison K, Patel DG, Greene L. Racial and ethnic disparities in primary open-angle glaucoma clinical trials: a systematic review and meta-analysis. *JAMA Netw Open*. 2021;4(5):e218348. doi:10.1001/jamanetworkopen.2021.8348

**eFigure.** Study Selection Process Using the PRISMA Format

**eTable 1.** Search Terms Used

**eTable 2.** Characteristics of Glaucoma Treatments in POAG Clinical Trials

**eTable 3.** Types of Sponsors

This supplementary material has been provided by the authors to give readers additional information about their work.

© 2021 Allison K et al. *JAMA Network Open.*

**eFigure.** Study Selection Process Using the PRISMA Format

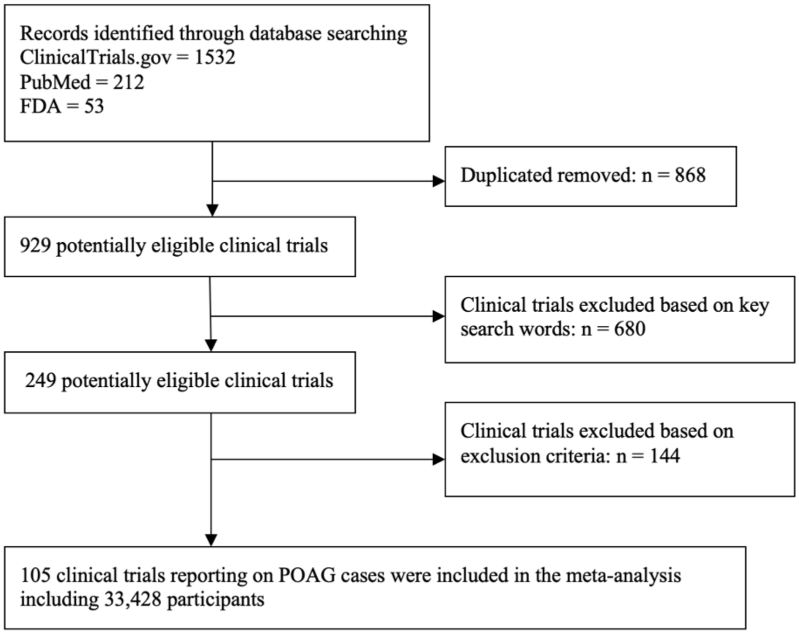


**eTable 1.** Search Terms Used

| Search Terms | Combinations | Filters |
|---|---|---|
| Glaucoma, primary open angle | "studies with results" OR | Completed |
| | "interventional studies (clinical trials)" OR | Publicly available with results |
| | | IRB approved |
| Primary open-angle glaucoma | "therapy" OR | Completed |
| | | Publicly available with results |
| | | IRB approved |

© 2021 Allison K et al. *JAMA Network Open.*

**eTable 2.** Characteristics of Glaucoma Treatments in POAG Clinical Trials

| **Medical Intervention** | **# of Trials** | **Total Population, N** | **White, n (%)** | **Black, n (%)** | **Other, n (%)** | **Hispanic/ Latino, n (%)** | **Female, n (%)** | **Male, n (%)** |
|---|---|---|---|---|---|---|---|---|
| Drug | 89 | 30449 | 21204 (69.6) | 5324 (17.5) | 2859 (9.4) | 1062 (3.5) | 16963 (55.7) | 13486 (44.3) |
| Device | 10 | 1275 | 1014 (79.5) | 105 (8.2) | 98 (7.7) | 58 (4.5) | 695 (54.5) | 580 (45.4) |
| Procedure | 6 | 1704 | 1418 (83.2) | 183 (10.7) | 81 (4.8) | 22 (1.3) | 746 (43.8) | 958 (56.2) |
| **Total** | 105 | 33428 | 23636 (70.7) | 5612 (16.8) | 3038 (9.1) | 1142 (3.4) | 18404 (55.1) | 15024 (44.9) |

© 2021 Allison K et al. *JAMA Network Open.*

**eTable 3.** Types of Sponsors

| **SPONSORS** | |
|---|---|
| US PHARMACEUTICAL COMPANIES | # of Clinical Trials |
| Pfizer | 7 |
| Oak Pharmaceuticals, Inc. | 6 |
| Glaukos Corporation | 3 |
| Merck Sharp and Dohme Corporation | 2 |
| Aerie Pharmaceuticals | 19 |
| Nephron Pharmaceuticals Corporation | 1 |
| Inotek Pharmaceutical Corporation | 1 |
| Actavis, Inc. | 1 |
| NON-US PHARMACEUTICAL COMPANIES | # of Clinical Trials |
| Allergan | 16 |
| Novartis | 13 |
| Santen Pharmaceutical Company, Ltd. | 3 |
| Transcend Medical | 2 |
| Bausch and Lomb, Inc. | 8 |
| Ono Pharmaceuticals USA, Inc. | 3 |
| AqueSys, Inc. | 1 |
| ForSight Vision 5, Inc. | 2 |
| Shire | 1 |
| NON-PHARMACEUTICAL COMPANIES | # of Clinical Trials |
| Washington University School of Medicine + National Eye Institute | 1 |
| Azienda Socio Sanitaria Territoriale degli Spedali Civili di Brescia | 1 |
| Massachusetts Eye and Ear Infirmary | 1 |
| Wills Eye | 3 |
| Eye Tech Care | 1 |
| Sensimed AG | 1 |
| ScienceBased Health | 1 |
| National Institute for Health Research | 1 |
| University of Sao Paulo (Conselho Nacional de Desenvolvimento Cientifico e Tecnologico) | 1 |
| Envisia Therapeutics | 1 |
| University of Colorado – Denver | 1 |
| Ivantis, Inc. | 1 |
| COLLABORATORS | # of Clinical Trials |
| Japan Association of Health Service + Alcon Japan | 1 |
| Cornerstone Health Care, PA + Allergan | 1 |

© 2021 Allison K et al. *JAMA Network Open.*
